# Two Multiplex Real-Time PCR Assays to Detect and Differentiate *Acinetobacter baumannii* and Non- *baumannii Acinetobacter* spp. Carrying *bla*_NDM_, *bla*_OXA-23-Like_, *bla*_OXA-40-Like_, *bla*_OXA-51-Like_, and *bla*_OXA-58-Like_ Genes

**DOI:** 10.1371/journal.pone.0158958

**Published:** 2016-07-08

**Authors:** Qiu Yang, Yongyu Rui

**Affiliations:** Laboratory Medicine Center, Nanfang Hospital, Southern Medical University, Guangzhou, China; University of Malaya, MALAYSIA

## Abstract

Nosocomial infections caused by *Acinetobacter* spp. resistant to carbapenems are increasingly reported worldwide. Carbapenem-resistant *Acinetobacter* (CRA) is becoming a serious concern with increasing patient morbidity, mortality, and lengths of hospital stay. Therefore, the rapid detection of CRA is essential for epidemiological surveillance. Polymerase chain reaction (PCR) has been extensively used for the rapid identification of most pathogens. In this study, we have developed two multiplex real-time PCR assays to detect and differentiate *A*. *baumannii* and non-*A*. *baumannii Acinetobacter* spp, and common carbapenemase genes, including *bla*_NDM_, *bla*_OXA-23-like_, *bla*_OXA-40-like_, *bla*_OXA-51-like_, and *bla*_OXA-58-like_. We demonstrate the potential utility of these assays for the direct detection of *bla*_NDM_-, *bla*_OXA-23-like_-, *bla*_OXA-40-like_-, *bla*_OXA-51-like_-, and *bla*_OXA-58-like_-positive CRA in clinical specimens. Primers were specifically designed, and two multiplex real-time PCR assays were developed: multiplex real-time PCR assay1 for the detection of *Acinetobacter baumannii* 16S–23S rRNA internal transcribed spacer sequence, the *Acinetobacter recA* gene, and class-B-metalloenzyme-encoding gene *bla*_NDM_; and multiplex real-time PCR assay2 to detect class-D-oxacillinase-encoding genes (*bla*_OXA-23-like_, *bla*_OXA-40-like_, *bla*_OXA-51-like_,and *bla*_OXA-58-like_). The assays were performed on an ABI Prism 7500 FAST Real-Time PCR System. CRA isolates were used to compare the assays with conventional PCR and sequencing. Known amounts of CRA cells were added to sputum and fecal specimens and used to test the multiplex real-time PCR assays. The results for target and nontarget amplification showed that the multiplex real-time PCR assays were specific, the limit of detection for each target was 10 copies per 20 μL reaction volume, the assays were linear over six log dilutions of the target genes (r^2^ > 0.99), and the Ct values of the coefficients of variation for intra- and interassay reproducibility were less than 5%. The multiplex real-time PCR assays showed 100% concordance with conventional PCR when tested against 400 CRA isolates and their sensitivity for the target DNA in sputum and fecal specimens was 10^2^ CFU/mL. Therefore, these novel multiplex real-time PCR assays allow the sensitive and specific characterization and differentiation of *bla*_NDM_-, *bla*_OXA-23-like_-, *bla*_OXA-40-like_-, *bla*_OXA-51-like_-, and *bla*_OXA-58-like_-positive CRA, making them potential tools for the direct detection of CRA in clinical specimens and the surveillance of nosocomial infections.

## Introduction

The genus *Acinetobacter* comprises strictly aerobic, Gram-negative, non-fermenting coccobacilli, which have become some of the most prominent human pathogens, causing a wide range of nosocomial infections [1]. Thirty-four species have been identified within this genus [2]. *Acinetobacter* spp. can colonize patients or the equipment used in medical care and survive on environmental surfaces for prolonged times [3]. Most clinical isolates are reported to be strains of *Acinetobacter baumannii* [4]. However, clinical infections caused by non-*baumannii Acinetobacter* spp. have increased [5]. In the last decade, carbapenem-resistant *Acinetobacter* (CRA) has become worldwide public-health issue with a widespread distribution, broad range of activities against β-lactams, increased patient morbidity, mortality, and lengths of hospital stay [6], particularly among elderly patients, infants and patients with severe underlying disease. Therefore, there is growing concern about the increasing prevalence of CRA [7], and it is difficult for clinicians to choose the initially appropriate antibiotic therapy [8].

Carbapenem resistance was first described in *A*. *baumannii* in the early 1990s and is most often mediated through carbapenem hydrolyzing enzymes [9]. The most common carbapenemases in *Acinetobacter* are the class D oxacillinases (OXA), including OXA-23-like, OXA-40-like, OXA-51-like, and OXA-58-like enzymes [10]. In recent years, carbapenemases from classes B have also been involved[7]. New Delhi metallo-β-lactamase 1 (NDM-1), a novel metallo-β-lactamase, was first reported in India in 2008 [11], and is a rapidly emerging resistance gene of the family *Enterobacteriaceae* [7]. However, according to two recent large-scale polymerase chain reaction (PCR)-based surveillance programs for NDM-1 in China [12, 13], *Acinetobacter* is the primary *bla*_NDM-1_-positive genus rather than members of the Enterobacteriaceae.

In the past few decades, conventional PCR, real-time PCR, and loop-mediated isothermal amplification (LAMP) have been used extensively to rapidly identify most pathogens. *A*. *baumannii* and class D oxacillinase genes have been detected simultaneously with multiplex conventional PCR [14, 15],however, conventional PCR required openning the tubes for procedures post detection during the reaction which might cause contamination and lead to false-positive results [16]. Therefore,the use of real-time PCR and LAMP is attractive, because they are highly sensitive and specific, time efficient, and generate fewer false-positive results. As we known,the LAMP method is difficult to apply in a multiplex assay because six primers are used to detect one target sequence [17], while probe-based and dye-based multiplex real-time PCR assays have been used to detect several targets simultaneously. Fluorescently labeled target-specific probes can add significant costs to an assay,however,intercalating dyes such as SYBR Green I have been reported suitable for multiplex real-time PCR systems, it detect all double stranded DNA, and PCR products can be distinguished by their melt curves [18–22].

The aim of this study was to establish and evaluate two multiplex real-time PCR assays that incorporate the primers for seven target genes, followed by a melting-curve analysis of the amplicons, to simultaneously detect and differentiate the *A*. *baumannii* 16S–23S rRNA internal transcribed spacer (ITS), the *Acinetobacter recA* gene, and the class-B-metalloenzyme-encoding gene *bla*_NDM_ with multiplex real-time PCR assay 1, and the class-D-oxacillinase-encoding genes(*bla*_OXA-23-like_, *bla*_OXA-40-like_, *bla*_OXA-51-like_,and *bla*_OXA-58-like_) in multiplex real-time PCR assay 2.

## Materials and Methods

### Bacterial strains and antibiotic susceptibility testing

A total of 400 carbapenem-resistant (with resistance to imipenem or meropenem) *Acinetobacter* were isolated at Nanfang Hospital in Guangzhou, southern China. Three hundred and fifty of them were collected from different clinical specimens from diverse units of the hospital, between January 2010 and December 2014, and the other 50 were collected from the stool specimens of inpatients, between January 2014 and December 2014. Species identification and antimicrobial susceptibility testing were performed with the BD Phoenix 100 Automated Microbiology System (Becton, Dickinson and Co., Franklin Lakes, NJ, USA). The results of susceptibility testing were interpreted according to the Clinical and Laboratory Standards Institute (CLSI) guidelines (CLSI-M100-S23). The isolates were stored at –80°C in nutrient broth containing 30% (v/v) glycerol.

### DNA extraction

Fresh well-isolated colonies were used for total bacterial DNA extraction with a Mag-MK Bacterial Genomic DNA extraction kit (Sangon Biotech, Shanghai, China), according to the protocol of the manufacturer. After the final DNA extraction step, the DNA was dissolved in 40 μL of Tris-EDTA buffer and stored at –20°C.

### Primer design

The details of the reference genes used in the assays were obtained from the NCBI homepage (http://www.ncbi.nlm.nih.gov/). These genes were: *A*. *baumannii* 16S–23S rRNA ITS (the ITS region between the 16S and 23S rRNA genes is a good candidate for bacterial species identification [23]; *Acinetobacter recA* gene, which was used as the control for the genus *Acinetobacter* [24]; class-B-metalloenzyme-encoding gene *bla*_NDM_ [25], and the class-D-oxacillinase-encoding genes(*bla*_OXA-23-like_, *bla*_OXA-40-like_, *bla*_OXA-51-like_,and *bla*_OXA-58-like_)[9]. The sequences of these genes were obtained from GenBank homepage (http://www.ncbi.nlm.nih.gov/GenBank). Based on comprehensive analysis and alignment of each gene type, primers were specifically designed to amplify all the alleles of each gene (except *bla*_OXA-51-like_) described above. The melting temperature (T_m_) of the amplification product of each carbapenemase gene family member was determined with the Beacon Designer software (Premier Biosoft, Palo Alto, CA, USA) (Table 1). All the primers were synthesized by Sangon Biotech Co. Ltd.

**Table 1:** PCR primers of the multiplex real-time PCR assays.

Primer	Sequence(5^,^-3^,^)	position	Aplicom size	Tm value	Reference
16S-23S rRNA ITS	CATTATCACGGTAATTAGTG	343–362	208	78.7	AY601823
	AGAGCACTGTGCACTTAAG	532–550			
recA	CCTGAATCTTCTGGTAAAAC	598–617	405	84.2	L26100
	GTTTCTGGGCTGCCAAACATTAC	1000–1022			
NDM	GCATTAGCCGCTGCATTGAT	46–65	828	91.2	KC310727
	GCCCCCTGTCACATCGAAAT	854–873			
OXA-23-like	TTTACTTGCTATGTGGTTGCT	13–33	107	77.5	KC310727
	ATCACCTGATTATGTCCTTGA	99–119			
OXA-24-like	GCACCTATGGTAATGCTCT	191–209	185	79.2	AJ239129
	CATTGCCTCACCTAAAGTC	357–375			
OXA-51-like	AGTGAAGCGTGTTGGTTAT	441–459	285	81.6	KJ584925
	CAGCCTACTTGTGGGTYTA	671–689			
OXA-58-like	GCCTTTGACAATTACACCT	516–534	311	83.4	KF700121
	AACCCAACTTATCTAGCACAT	806–826			

### Simplex real-time PCR assays

To confirm the specificity of the real-time PCR assays, the primers were evaluated in a single SYBR-Green-I-based real-time PCR to ensure that the amplicons showed the expected T_m_ values. The amplicons were then analyzed with electrophoresis on a 2% agarose gel.

### Recombinant plasmids

Amplification products of 16S–23S rRNA ITS, recA, *bla*_NDM-1_, *bla*_OXA-23_, *bla*_OXA-40_, *bla*_OXA-51_, and *bla*_OXA-58_ genes were cloned into the plasmid pMD18-T (TaKaRa Bio Inc., Dalian, China),respectively, and then sequenced by TaKaRa. Recombinant standard plasmid p16S–23S rRNA ITS, precA, pNDM-1, pOXA-23, pOXA-40, pOXA-51, and pOXA-58 was constructed to optimize the quantitative PCR (qPCR).

### Multiplex real-time PCR assays

In multiplex real-time PCR 1, primers specific for the p16S–23S rRNA ITS-, precA-, and pNDM-1-positive strains were combined. In multiplex real-time PCR 2, primers specific for the pOXA-23-, pOXA-40-, pOXA-51-, and pOXA-58-positive strains were combined. The SYBR-Green-I based real-time PCR assays were performed on an ABI Prism 7500 FAST apparatus (Applied Biosystems, Foster City, CA, USA) using the SYBR Premix Ex Taq™ kit (TaKaRa Bio Inc., Dalian, China), as recommended by the manufacturers. The qPCR mixture contained 1 μL of extracted DNA, 10 μL of SYBR Premix Ex Taq qPCR (2×), 0.4 μL of ROX Reference Dye II (50×), pairs of primers (optimized to final concentrations of 0.2 mM for Ab16S–23S rRNA ITS, to 0.05 mM for recA, and 0.025 mM for NDM in multiplex real-time PCR 1; the primers were optimized to 0.075 mM for OXA-23-like, 0.05 mM for OXA-40-like, 0.05 mM for OXA-51-like, and 0.025 mM for OXA-58-like in multiplex real-time PCR 2), and sterile water to a final reaction volume of 20 μL. Each run was performed with the seven positive controls and a water blank as the negative control. The optimal cycling conditions were 5 min at 95°C; 35 cycles of 20 s at 95°C, 45 s at 55°C, and 30 s at 72°C; and a melting curve step, gradually increasing from 65°C by 0.1°C /s to 95°C (with fluorescence data acquisition every 1s). All real-time reactions were performed in triplicate.

### Standard curve and sensitivity test on recombinant plasmids

To determine the efficiency of the multiplex real-time PCR assays, the Ct values obtained from a series of template DNA dilutions were graphed on the y axis versus the log of the dilution on the x axis. The slope of this line was used to determine the efficiency (E) according to the equation: E = 10^(–1/slope)^. Assay sensitivity and reproducibility were estimated with serial 10-fold dilutions of the recombinant plasmids p16S–23S rRNA ITS, precA, and pNDM-1 in multiplex real-time PCR 1, and pOXA-23, pOXA-40, pOXA-51, and pOXA-58 in multiplex real-time PCR 2.

### Detection of seven target genes in carbapenem-resistant *Acinetobacter* with multiplex real-time PCR and conventional PCR

Two hundred and twenty-eight of the 400 isolates were identified as the *A*. *calcoaceticus*–*A*. *baumannii* complex (ACB complex), since traditional identification methods are unable to differentiate *Acinetobacter pittii*,*A*. *nosocomialis*,*A*. *baumannii* and *A*. *calcoaceticus*,as they share similar phenotypic properties, therefore, in the clinical microbiology report, these species have been grouped together into the so-called ACB complex, according to BD Phoenix 100 Automated Microbiology System (Becton, Dickinson and Co., Franklin Lakes, NJ, USA). The ACB complex was further differentiated using the 16S–23S rRNA ITS sequence [23]. All the isolates were screened for the presence of *bla*_NDM_, *bla*_OXA-23-like_, *bla*_OXA-40-like_, *bla*_OXA-51-like_, and *bla*_OXA-58-like_ genes using conventional PCR and sequencing [15].

### Preparation of human specimens

Sputum and stool specimens were collected from inpatients after it had been confirmed that the samples were cultured without *Acinetobacter*, between July 2014 and December 2014. Ethical approval for the collection of patient specimens was obtained from the Medical Ethics Committee of NanFang Hospital (affiliated to Southern Medical University). Because sputum and stool specimens were collected after routine clinical work compeleted, informed consent was exempted as the Institutional Review Boards approved.To assess the sensitivity of the multiplex real-time PCR assays in detecting the target genes in sputum and stool specimens, serial dilutions of cultured *A*. *baumannii* 140721108 (*bla*_NDM-1_-carrying) cells, *A*. *baumannii* 158062 (*bla*_OXA-23_- and *bla*_OXA-51_-carrying) cells, non-*A*. *baumannii* 145509 (*bla*_*OXA*-40_-carrying) cells, non-*A*. *baumannii* 140821016 (*bla*_NDM-1_-carrying) cells, and non-*A*. *baumannii* 151128 (*bla*_OXA-58_-carrying) cells (10^1^–10^8^ CFU per mL) were prepared and 0.2 mL of each was added to the specimens. The sputum specimens were processed with the standard N-acetyl-l-cysteine (NALC)–sodium hydroxide (NaOH) digestion–decontamination method; an equal volume of 2% NaOH/1.45% sodium citrate containing 0.5% NALC was added to each specimens, mixed with vortexing, and then incubated at room temperature for 15 min. After the samples were shaken by hand at regular intervals, phosphate-buffered saline (pH 6.8) was added to 45 mL and then centrifuged at 3000 × *g* for 15 min. The supernatant was decanted, and the resulting sediment was resuspended in 1.8 mL of phosphate-buffered saline [26]. The fecal specimens (1 g) were suspended in 9 mL of distilled water with vigorous shaking for 5 min. The samples were allowed to precipitate by standing for 2 min, and 1.8 mL of the supernatants were used [27] to determine the final concentrations of CRA cells (10^0^–10^7^)CFU per mL. The DNA was extracted from these samples directly with the Mag-MK Bacterial Genomic DNA extraction kit (Sangon Biotech), according to manufacturer’s instructions.

## Results

### Specificity of the primers

**Fig 1:**
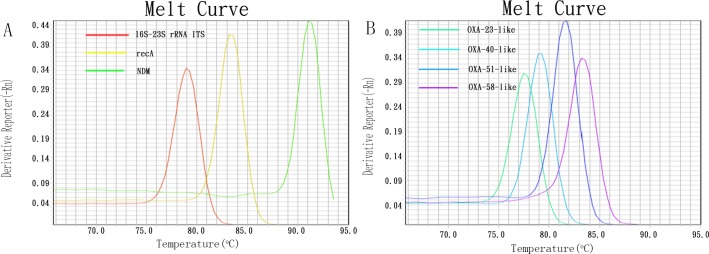
T_m_ value corresponding to seven DNA targets amplicon in real-time PCR. A: T_m_ value of Ab16S-23S rRNA ITS, recA and NDM amplicon,respectively;B: T_m_ value of OXA-23-like,OXA-40-like,OXA-51-like and OXA-58-like amplicon, respectively.

**Fig 2:**
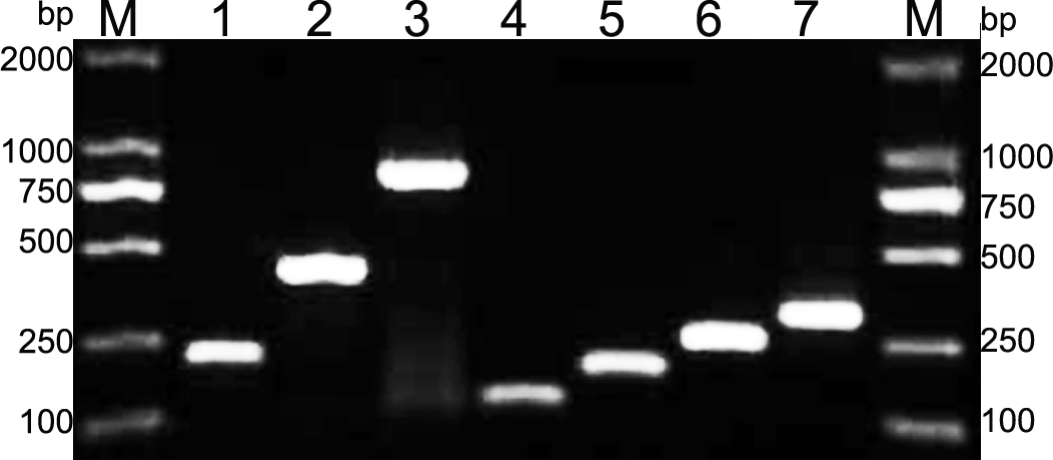
Agarose gel electrophoresis of seven primer sets conventional PCR products. M: DL2000 DNA marker 1:Ab16S-23S rRNA ITS; 2:recA; 3:NDM; 4:OXA-23-like; 5:OXA-40-like; 6:OXA-51-like; 7:OXA-58-like.

Primer specificities were evaluated at the NCBI homepage (http://www.ncbi.nlm.nih.gov). No matches to the primer sequences were found other than those to the corresponding genes. The primer pairs were initially validated using positive control strains carrying the target genes, identified as previously described [28]. Equivalent T_m_ values for each gene were detected when the positive control strains were tested with simplex real-time PCR assays. The T_m_ analysis of the amplicons provided the following results: Ab16S–23S rRNA ITS, T_m_ 78.7°C; *recA*, T_m_ 84.2°C; *bla*_NDM_ type, T_m_ 91.2°C; *bla*_OXA-23_ type, T_m_ 77.5°C; *bla*_OXA-40_ type, T_m_ 79.2°C; *bla*_OXA-51_ type, T_m_ 81.6°C; and *bla*_OXA-58_ type, T_m_ 83.4°C (Fig 1). The size of each PCR product was also confirmed by electrophoresis on a 2% agarose gel (Fig 2). Thus, the specificity of our multiplex real-time PCR assays was considered satisfactory.

### Establishment of multiplex real-time PCR assays

The novel multiplex real-time PCR assays were performed using real-time PCR and a melting-curve analysis. The assays were initially validated with recombinant plasmids carrying the target genes. The same T_m_ for each gene was detected when the positive control strains (p16S–23S rRNA ITS, precA, pNDM-1, pOXA-23, pOXA-40, pOXA-51, and pOXA-58) were tested with the multiplex real-time PCRassays.

**Fig 3:**
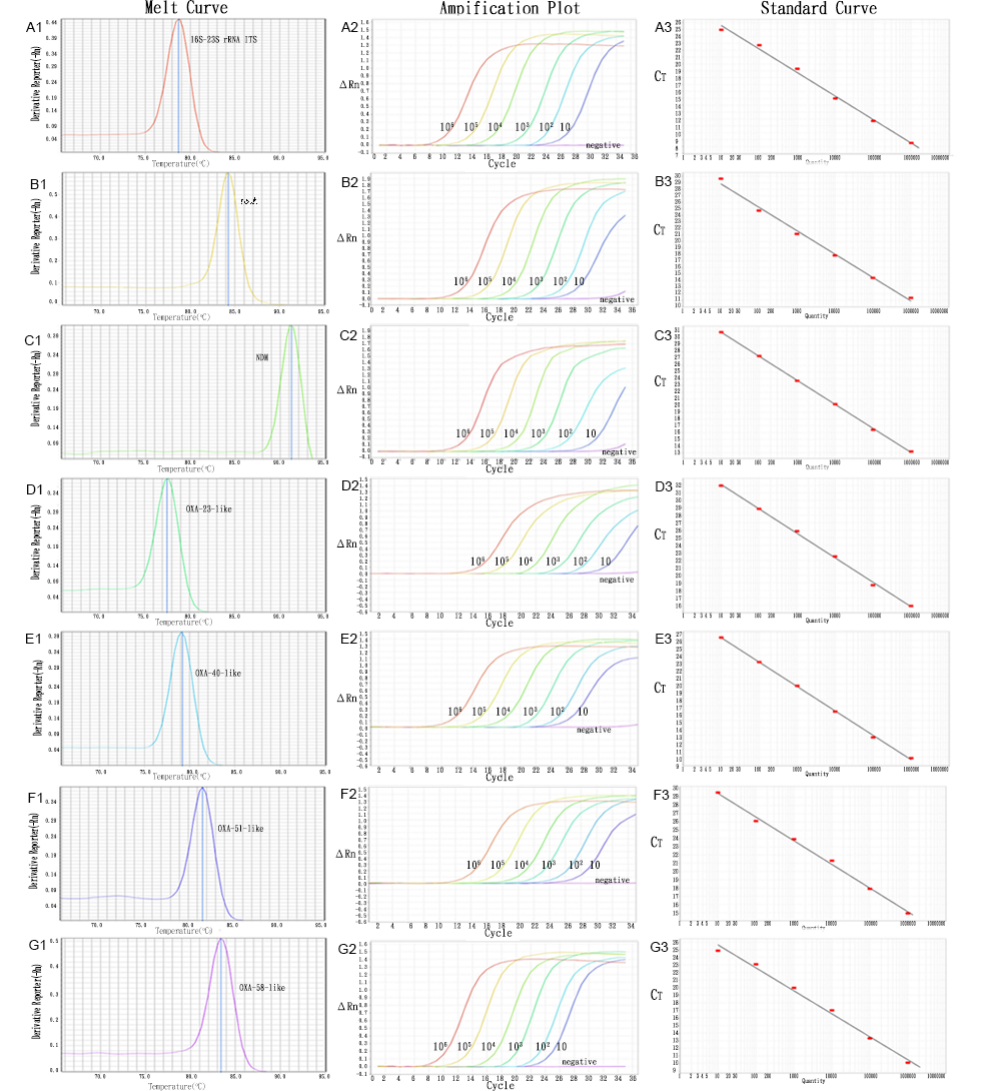
Specificity, amplification plots and standard curves of the multiplex real-time PCR assays. A:Ab16S-23S rRNA ITS; B:recA; C:NDM; D:OXA-23-like;E:OXA-40-like; F:OXA-51-like; G:OXA-58-like. A1-G1: specificity of the multiplex real-time PCR assays; A2-G2: amplification plots of 10-fold dilution series for recombination plasmids; A3-G3: standard curves of recombination plasmids.

The linearity and limits of detection of the assays were determined with serial 10-fold dilutions of each recombinant plasmid from 10 to 10^6^ copies/μL (Fig 3). The limit of detection for the target DNA was 10 copies per 20 μL reaction volume.The assays correlated well for p16S–23S rRNA ITS (r^2^ = 0.994), precA (r^2^ = 0.994), pNDM-1 (r^2^ = 1), pOXA-23 (r^2^ = 0.998), pOXA-40 (r^2^ = 0.999),pOXA-51 (r^2^ = 0.996), and pOXA-58 (r^2^ = 0.992) over the entire copy number range, with efficiencies of 0.962, 0.899, 0.909, 1.025, 0.991, 1.232, and 1.139, respectively.

We also showed that the assays were highly stable and precise, based on the performance of the recombinant plasmids. Intra- and interassay repeatability was tested in triplicate for each dilution within the same run and each concentration was repeated three different times to assess the reproducibility of the multiplex real-time PCR assays (S1, S2 and S3 Tables). The intra-assay variability for the Ct values corresponding to 10^4^ copies/L for each gene was low, with a coefficient of variation (CV) of 0.05%–0.46%. The interassay CV for the Ct values was also low, in the range of 0.05%–4.20% (Table 2). Intra- and interassay CVs of less than 4.2% confirmed the high repeatability of the assays.

**Table 2:** Reproducibility of the multiplex real-time PCR assays.

target	Intra -assay					Inter-assay				
	Ct of amplicons			Mean	CV%	Ct of amplicons			Mean	CV%
16S-23S rRNA ITS	24.96	25.12	25.00	25.03	0.34	25.03	25.94	25.05	25.34	2.05
recA	23.92	23.95	23.90	23.92	0.09	23.92	23.94	23.95	23.94	0.05
NDM	21.72	21.89	21.71	21.77	0.46	21.77	21.88	22.49	22.05	1.75
OXA-23-like	21.90	21.88	21.89	21.89	0.05	21.89	21.93	21.88	21.90	0.11
OXA-40-like	19.94	19.92	19.90	19.92	0.10	18.47	19.92	19.85	19.42	4.20
OXA-51-like	16.82	16.77	16.92	16.84	0.47	16.84	16.96	16.83	16.87	0.42
OXA-58-like	16.90	16.94	16.94	16.93	0.14	16.93	16.93	16.90	16.92	0.10

### multiplex real-time PCR assays compared with conventional PCR and sequencing for CRA isolates

**Fig 4:**
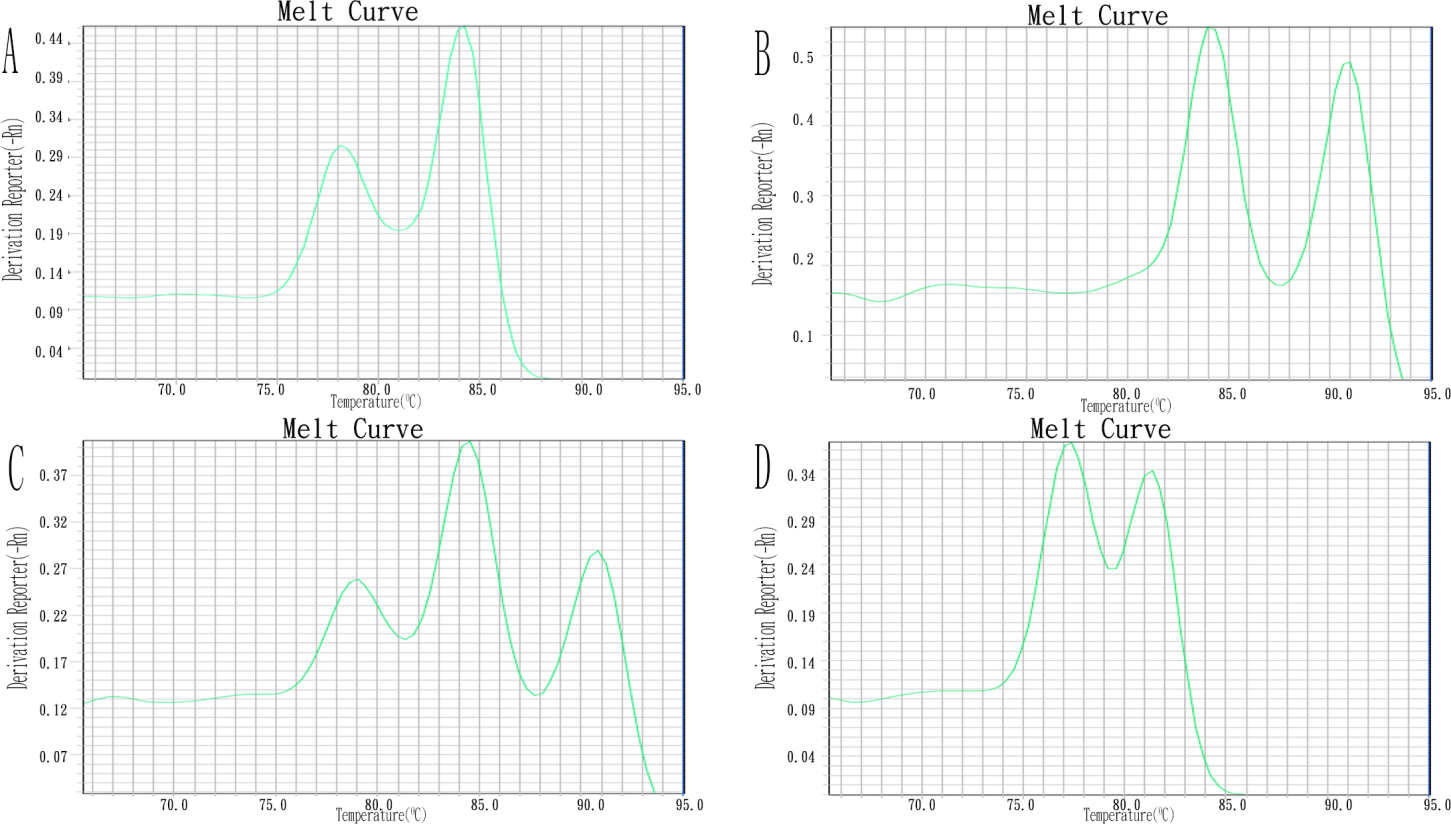
Representative results of the multiplex real-time PCR assays. A: *Acinetobacter baumannii* (co-detection of Ab16S-23S rRNA ITS and recA genes) in multiplex real-time PCR assay 1; B: non-*baumannii Acinetobacter* spp.carrying *bla*_NDM_(co-detection of recA and *bla*_NDM_ genes) in multiplex real-time PCR assay 1; C: *Acinetobacter baumannii* carrying *bla*_*NDM*_(co-detection of Ab16S-23S rRNA ITS,recA and *bla*_NDM_ genes) in multiplex real-time PCR assay 1; D: co-detection of *bla*_OXA-23-like_ and *bla*_OXA-51-like_ genes in multiplex real-time PCR assay 2.

When tested against 400 previously characterized CRA isolates, the multiplex real-time PCR assays showed 100% concordance with conventional PCR. The carbapenemase genes of these strains were confirmed with PCR and sequencing. The PCR amplification products were sequenced by the Beijing Genomics Institute (Shenzhen, China). (Table 3). When the multiplex real-time PCR assays were used, we rapidly characterized and differentiated *A*. *baumannii*, non-*A*. *baumannii*, and the carbapenemase genes carried by them by reading the T_m_ values from the melting curve (Fig 4).

**Table 3:** Results of 400 carbapenemas-resistant *Acinetobacter* via multiplex real-time PCR assays.

Gene types	*Acinetobacter baumannii*	non- *baumannii Acinetobacter* spp.
16S-23S rRNA ITS	228	0
recA	228	172
NDM	12	25
OXA-23-like	32	62
OXA-40-like	1	1
OXA-51-like	17	89
OXA-58-like	19	23
OXA-23-like and OXA-51-like	78	5

### Multiplex real-time PCR assays to directly detect CRA in human specimens

**Fig 5:**
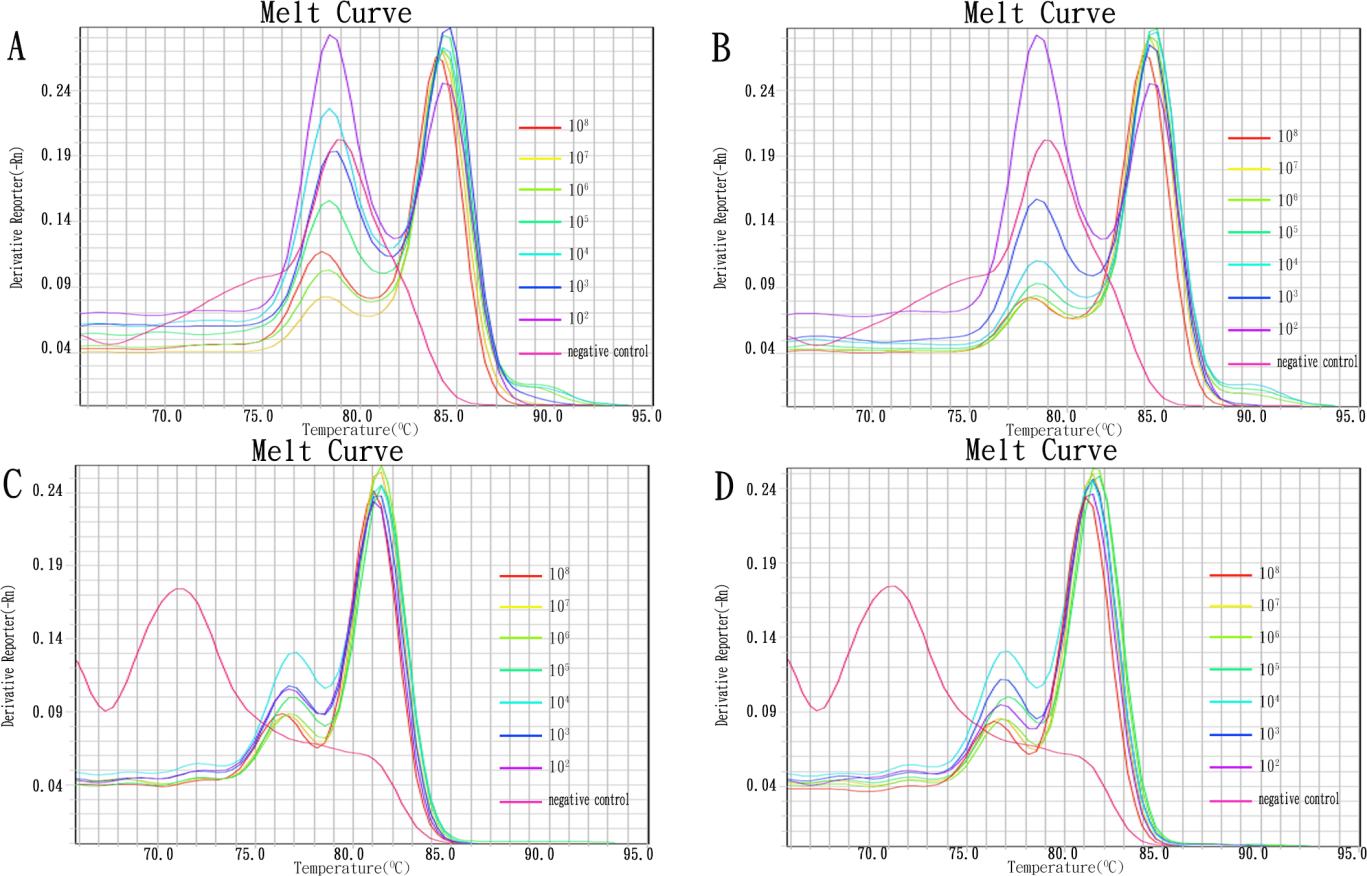
Representative results of the multiplex real-time PCR assays for directly detect target DNA from sputum and stool specimens. A: sensitivity of *Acinetobacter baumannii* (co-detection of Ab16S-23S rRNA ITSand recA genes) in multiplex real-time PCR assay 1 from sputum specimens; B: sensitivity of *Acinetobacter baumannii* (co-detection of Ab16S-23S rRNA ITS and recA genes) in multiplex real-time PCR assay 1 from stool specimens; C: sensitivity of carbapenemase genes (co-detection of *bla*_OXA-23-like_ and *bla*_OXA-51-like_ genes) in multiplex real-time PCR assay 2 from sputum specimens; D: sensitivity of carbapenemase genes (co-detection of *bla*_OXA-23-like_ and *bla*_OXA-51-like_ genes) in multiplex real-time PCR assay 2 from stool specimens.

We evaluated the sensitivity of the multiplex real-time PCR assays in directly detecting the 16S–23S rRNA ITS, *recA*, *bla*_NDM_, *bla*_OXA-23-like_, *bla*_OXA-40-like_, *bla*_OXA-51-like_, and *bl*a_OXA-58-like_ genes in sputum and fecal specimens. The sensitivity of the multiplex real-time PCR assays for the target DNAs in the sputum and fecal specimens was 10^2^ CFU/mL (Fig 5).

### Discussion

*Acinetobacter* spp. are known for their ability to colonize the nostrils, pharynx, skin, and rectum of patients and their remarkable capacity to upregulate or acquire resistance determinants [29]. In recent decades, *Acinetobacter* spp. have become a public-health issue because they play important roles in nosocomial infections [30]. The serious concern associated with these bacteria is the growing prevalence of carbapenem-resistant isolates. Therefore, surveillance and early detection are required to control these infections and prevent their further spread, so a sensitive and specific detection system for CRA is required. Although the multiplex real-time PCR technology has been extensively used for the rapid detection of pathogens, the SYBR-Green-I-based multiplex real-time PCR assays developed in this study are less expensive than probe-based multiplex real-time PCR assays [31]. Here, we have described the development and validation of two multiplex real-time PCR assays for CRA, and demonstrated their potential utility in the direct detection of tracheal and gastrointestinal colonization by *A*. *baumannii* and non-*baumannii Acinetobacter* spp. carrying the *bla*_NDM_, *bla*_OXA-23-like_, *bla*_OXA-40-like_, *bla*_OXA-51-like_, and *bla*_OXA-58-like_ genes in sputum and fecal specimens.

Our multiplex real-time PCR assays can detect almost all the alleles of each common carbapenemase gene (*bla*_NDM_, *bla*_OXA-23-like_, *bla*_OXA-40-like_, *bla*_OXA-51-like_, and *bla*_OXA-58-like_) of *Acinetobacter* spp., and can characterize and differentiate *A*. *baumannii* and non-*baumannii Acinetobacter* spp. The conventional PCR and sequencing method was used as the “gold standard”, and the multiplex real-time PCR assays showed 100% concordance when validated in 400 CRA isolates with well-defined carbapenemase genes. In our opinion, the 100% sensitivity and specificity of the multiplex real-time PCR assays against clinical isolates of CRA with well-defined common carbapenemase genes make it a useful tool for the screening and surveillance of *Acinetobacter* isolates carrying the carbapenemase genes *bla*_NDM_, *bla*_OXA-23-like_, *bla*_OXA-40-like_, *bla*_OXA-51-like_, and *bla*_OXA-58-like_.

The magnetic bead method was used for DNA extraction because it has the highest extraction efficiency and lowest relative loss ratio of the three DNA isolation methods [32]. Clinical samples, such as blood, urine, stools, and sputum, contain inhibitors that can interfere with PCR, causing amplification failure and increasing the false-negative results. Of all of these samples, sputum and stools are likely to contain the most diverse inhibitory factors because of their heterogeneity and complexity [33]. In this study, we added serial dilutions of cultured CRA cells to human sputum and fecal specimens in the experiments. The sensitivity of the multiplex real-time PCR assays for the target DNA in the sputum and fecal specimens was 10^2^ CFU/mL, which is similar to previously published data [34, 35].

## Conclusion

In conclusion, the multiplex real-time PCR assays provide low-cost, sensitive, and specific characterization and differentiation for most of the CRA, and making them potential tools for the direct detection of CRA in clinical specimens.

## Supporting Information

S1 Table Raw data of reproducibility of the multiplex real-time PCR assays 1. (XLS)

S2 Table Raw data of reproducibility of the multiplex real-time PCR assays 2. (XLS)

S3 Table Raw data of reproducibility of the multiplex real-time PCR assays 3. (XLS)
